# Nonclassical monocytes potentiate anti-tumoral CD8^+^ T cell responses in the lungs

**DOI:** 10.3389/fimmu.2023.1101497

**Published:** 2023-06-22

**Authors:** Lindsey E. Padgett, Paola M. Marcovecchio, Claire E. Olingy, Daniel J. Araujo, Kathleen Steel, Huy Q. Dinh, Ahmad Alimadadi, Yanfang Peipei Zhu, Melissa A. Meyer, William B. Kiosses, Graham D. Thomas, Catherine C. Hedrick

**Affiliations:** ^1^ Division of Inflammation Biology, La Jolla Institute for Allergy and Immunology, La Jolla, CA, United States; ^2^ Center for Autoimmunity and Inflammation, La Jolla Institute for Immunology, La Jolla, CA, United States

**Keywords:** monocytes, nonclassical monocytes, cancer, metastasis, CD8 lymphocytes

## Abstract

CD8^+^ T cells drive anti-cancer immunity in response to antigen-presenting cells such as dendritic cells and subpopulations of monocytes and macrophages. While CD14^+^ classical monocytes modulate CD8^+^ T cell responses, the contributions of CD16^+^ nonclassical monocytes to this process remain unclear. Herein we explored the role of nonclassical monocytes in CD8^+^ T cell activation by utilizing E2-deficient (E2^-/-^) mice that lack nonclassical monocytes. During early metastatic seeding, modeled by B16F10-OVA cancer cells injected into E2^-/-^ mice, we noted lower CD8^+^ effector memory and effector T cell frequencies within the lungs as well as in lung-draining mediastinal lymph nodes in the E2^-/-^ mice. Analysis of the myeloid compartment revealed that these changes were associated with depletion of MHC-II^lo^Ly6C^lo^ nonclassical monocytes within these tissues, with little change in other monocyte or macrophage populations. Additionally, nonclassical monocytes preferentially trafficked to primary tumor sites in the lungs, rather than to the lung-draining lymph nodes, and did not cross-present antigen to CD8^+^ T cells. Examination of the lung microenvironment in E2^-/-^ mice revealed reduced CCL21 expression in endothelial cells, which is chemokine involved in T cell trafficking. Our results highlight the previously unappreciated importance of nonclassical monocytes in shaping the tumor microenvironment via CCL21 production and CD8^+^ T cell recruitment.

## Introduction

CD8^+^ T cells embody a crucial component of the anti-tumorigenic immune response. Indeed, cytotoxic and memory CD8^+^ T cells possess strong prognostic value for survival in a wide variety of cancers (1, 2). The presence of effector memory CD45RO^+^ T cells is positively associated with metastasis-free tumors and a positive clinical outcome in colorectal cancer (3). Moreover, enhanced cytotoxic CD8^+^ T cell frequencies within the tumor microenvironment (TME) are correlated with favorable outcomes in breast (4, 5), colorectal (6), melanoma (7), ovarian (8), and pancreatic cancers (9). Myeloid cells, such as CD103^+^ DCs and CD169^+^ subcapsular macrophages, prime CD8^+^ T cells in tumor-draining lymph nodes (LNs) by presenting processed tumorigenic peptides, a critical initial step for generating an anti-tumoral immune response (10–12). However, in contrast to DCs and macrophages, the interplay between monocytes and T cells remains largely uncharacterized.

Originating in the bone marrow, monocytes migrate to the blood, where they circulate for several days (13, 14). Blood monocytes are broadly divided into 2 major populations in mice: Ly6C^hi^CCR2^hi^CX3CR1^lo^ classical monocytes (cMo) and Ly6C^lo^CCR2^lo/-^CX3CR1^hi^ nonclassical monocytes (nMo) (15), with an intermediate population (iMo) making up a smaller subset (16). In contrast to cMo, nMo remain longer in circulation and patrol the vasculature as sentinels via engulfing apoptotic endothelial cells (16). nMo are widely believed to arise from differentiation of their classical monocyte counterparts and their differentiation and/or survival is controlled by the nuclear orphan receptor Nr4a1 (16–20).

Following influenza virus infection, cMo are the dominant antigen-presenting cells within the draining lymph nodes and elicit robust pro-inflammatory immune responses (21). Ly6C^hi^ cMo can present antigen, induce T follicular helper cell differentiation via IL-6 (22), and influence T cell responses by secreting chemokines that attract distinct T cell types. Specifically, tumor-embedded Ly6C^hi^ monocytic cells have been shown to secrete CCL3, CCL4, and CCL5, which recruit immunosuppressive T regulatory cells (Tregs) in a CCR5-dependent manner (23). In clinical studies, the presence of cMo in the tumor inversely correlates with CD8^+^ T cells. Similarly, CCL2 blockade reduces cMo and increases CD8^+^ T cell infiltration in tumors. Moreover, tumors with low CD8^+^ T cell infiltration and high CCL2 expression are associated with an unfavorable cancer prognosis (24).

In contrast to cMo, very little is known about how nMo may influence CD8+ T cell responses and the accompanying impact on anti-tumorigenic immunity. Previous studies by our laboratory demonstrated that nMo phagocytize tumor antigens via CX3CR1 and subsequently recruit anti-tumoral natural killer cells, resulting in tumor killing (25). A major limitation of these studies was a reliance on a full body depletion of *Nr4a1*, thus affecting other cell types, including macrophages. To mitigate these effects, our laboratory generated a mouse strain that specifically lacks nonclassical monocytes by depletion of the *Nr4a1* E2 super enhancer subdomain, resulting in targeted depletion of nonclassical monocytes, while simultaneously preserving classical monocyte frequencies and macrophage function. These mice (henceforth referred to as E2^-/-^) display markedly increased formation of lung metastases in both melanoma and lung cancer models (25–27), which partially depends on NK cells (27). As CD8+ T cells play a critical role in limiting tumor growth, and very little is known about the influence of nMo on these responses, here we sought to examine the role of Ly6C^lo^ nMo on T cell phenotypes and levels. To accomplish this, we characterized antigen-specific T cell responses to B16F10-OVA in E2^-/-^ mice lacking nMo. In this murine model, we found that effector memory CD8^+^ T cells were severely reduced in number and naive CD8^+^ T cell frequencies were increased in the lung after early lung seeding (6 days) by B16F10-OVA melanoma cells. Analysis of E2^-/-^ lungs and lung-draining lymph nodes revealed loss of memory CD8^+^ T cell responses. Examination of the lung microenvironment indicated a profound diminution of the chemokine CCL21, which modulates lymphocyte recruitment. These results identify a previously undescribed role for nMo in modulating T cell phenotypes by controlling the metastatic tumor microenvironment.

## Results

### Ly6C^lo^MHC-II^lo^ monocytes are absent in the lungs and tumor-draining lymph nodes of E2^-/-^ mice

**Figure 1:**
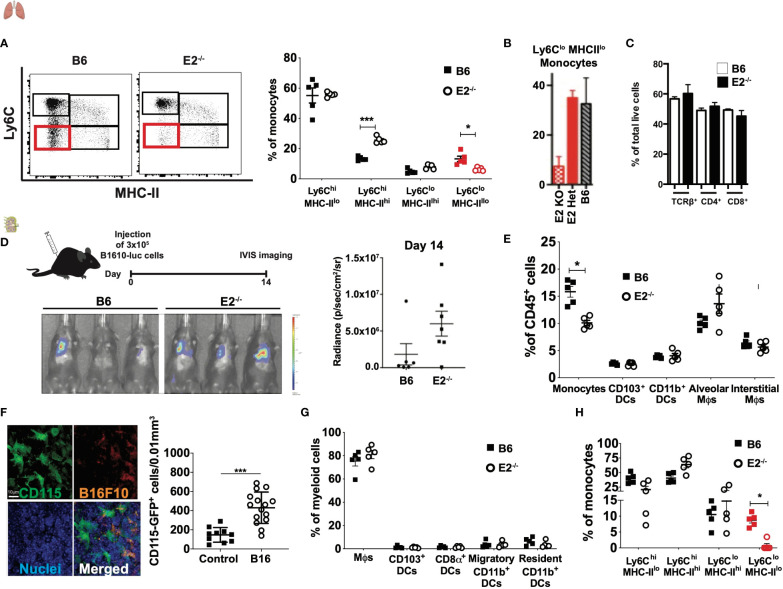
E2^-/-^ mice display loss of Ly6CloMHC-II^lo^ monocytes in the lungs and lymph nodes with normal T cell frequencies. Representative dot plots of Ly6C by MHC-II, with Ly6C^lo^MHC-II^lo^ populations in lung enumerated in red. Pooled frequencies of Ly6C^hi^MHC-II^hi^, Ly6C^hi^MHC-II^lo^, Ly6C^lo^MHC-II^hi^, Ly6C^lo^MHC-II^lo^ frequencies within lungs of B6 and E2^-/-^ mice at 6 days post B16F10-OVA injection **(A)**. Total monocyte frequencies as a percentage of CD115^+^ cells at baseline in blood from wild-type (WT), E2 heterozygous (Het) and homozygous (KO) mice **(B)**. T cell frequencies at baseline in mediastinal lymph nodes of E2^-/-^ mice **(C)**. Increased metastasis of cancer cells to lung in E2^-/-^ mice at day 14 after injection of B16F10-luciferase tumor cells **(D)**. Pooled frequencies of monocytes, CD103+ DCs, CD11b+ DCs, alveolar macrophages (Mϕs), and interstitial Mϕs of lungs at 6 days post injection of B16F10-OVA tumor cells **(E)**. Confocal microscopy of CD115 (as measured by GFP), B16F10 (as measured by RFP) and nuclei within MacGreen mice at 8h post-injection with B16F10-RFP cells, and pooled frequencies of CD115-GFP+ cells within control (n=10) and B16F10-RFP-injected (n=15) recipients **(F)**. Pooled frequencies of Mϕs, CD103+ DCs, CD8a+ DCs, migratory CD11b+ DCs, and resident CD11b+ DCs in B6 and E2^-/-^ mediastinal lymph nodes **(G)**. Pooled frequencies of Ly6C^hi^MHC-II^hi^, Ly6C^hi^MHC-II^lo^, Ly6C^lo^MHC-II^hi^, Ly6C^lo^MHC-II^lo^ (in red) monocytes in medLNs of B6 (n=5) and E2^-/-^ (n=5) mice at 6 days post injection with B16F10-OVA **(H)**. Results are expressed as mean + s.e.m. from at least two independent experiments within B6 and E2^-/-^ mice. Statistically significant differences were at *p < 0.05 or ***p<0.01; (One-way ANOVA).

A previous study by our laboratory found that nonclassical monocytes (nMo) engulf tumor material and recruit natural killer (NK) cells to lung to prevent lung tumor metastasis (25, 27). We have previously reported the generation of the E2^-/-^ mouse, which lacks a 1500bp enhancer upstream of the *Nr4a1* gene (26). The E2^-/-^ mice have a significant loss of nMo (Ly6C^lo^MHC-II^lo^) both after tumor seeding (**Figure 1A**; see **Supplemental Figure 1A** for gating strategy) and at baseline (**Figure 1B**, (26)). T cell (**Figure 1C**) and macrophage (26) frequencies are normal at baseline. CD4 and CD8 T cell frequencies are normal in lung and lung-draining lymph nodes after tumor seeding (**Supplementary Figure 1B**). The E2-/- mice showed increased metastasis of cancer cells to lung (**Figure 1D**, (26)). Here, we investigated the selective loss of nMo on regulating CD8 T cell responses in lung and lymph nodes in the setting of cancer.

First, we examined frequencies of known myeloid populations within the lung in E2^-/-^ mice lacking nMo after tumor seeding. After B16F10-OVA tumor cell injection, lungs from both B6 wildtype and E2^-/-^ mice possessed similar frequencies of CD103^+^ DCs, CD11b^+^ DCs, alveolar and interstitial macrophages (**Figure 1E**), with a concomitant 50-60% loss of total lung monocytes due to loss of nMo (26) (**Figure 1A** and **Supplemental Figure 1A**).

Here, we first determined if monocytes were enriched in the lung-draining medLNs during cancer metastasis to the lung in control mice. For this experiment, RFP-labeled B16F10 cancer cells were injected intravenously into MacGreen recipient mice that express eGFP under the control of the ​*Csf1r*​ promoter. Examination of medLN by confocal microscopy revealed robust expression of CD115 with a 2.9-fold increase in CD115-GFP+​​cells in the medLNs 8 hrs following B16F10-RFP cancer cell injection (​**Figure 1F**). Collectively, these results indicate that monocytes are increased in frequency in the medLN in metastatic cancer.

Next, to determine if myeloid cell populations in the lung-draining lymph nodes (LN) were perturbed by nMo deficiency in the setting of cancer, we analyzed the myeloid compartment within the mediastinal LN (medLNs) via flow cytometry (**Supplemental Figure 1C**) after tumor cell seeding. Within this LN compartment, dendritic cells (DCs) are commonly classified as resident CD8α^+^ and CD11b^+^ DCs or migratory CD103^+^ and CD11b^+^ DCs (12). Migratory CD103^+^ DCs can present cancer antigens in the LNs and are potent stimulators of CD8^+^ T cells. CD103^+^ DCs can also recruit CXCR3^+^ T_Eff_ cells by secreting the pro-inflammatory chemokines CXCL9 and CXCL10 (12, 28). After B16F10-OVA injection, E2^-/-^ mice possessed similar frequencies of CD115^+^CD11b^+^ monocytes, macrophages, and DCs (including migratory CD103^+^ DCs, resident CD8α^+^ DCs, migratory CD11b^+^ and resident CD11b^+^ DC populations in LN to those observed in control mice (**Figure 1G**). We observed loss of MHC-II^lo^Ly6C^lo^ monocytes in the medLN of E2^-/-^ mice compared to B6 mice after B16F10-OVA injection, despite similar frequencies of Ly6C^hi^MHC-II^hi^, Ly6C^hi^MHC-II^lo^, and Ly6C^lo^MHC-II^hi^ cells in both groups (**Figure 1H**). Together, these results indicate that Ly6C^lo^MHC-II^lo^ monocytes are depleted in the lungs and draining lymph nodes of E2^-/-^ mice in metastatic cancer.

### E2^-/-^ mice lacking nonclassical monocytes showed reduced CD8^+^ T cell activation and memory states in lung-draining lymph nodes after early tumor cell seeding

**Figure 2:**
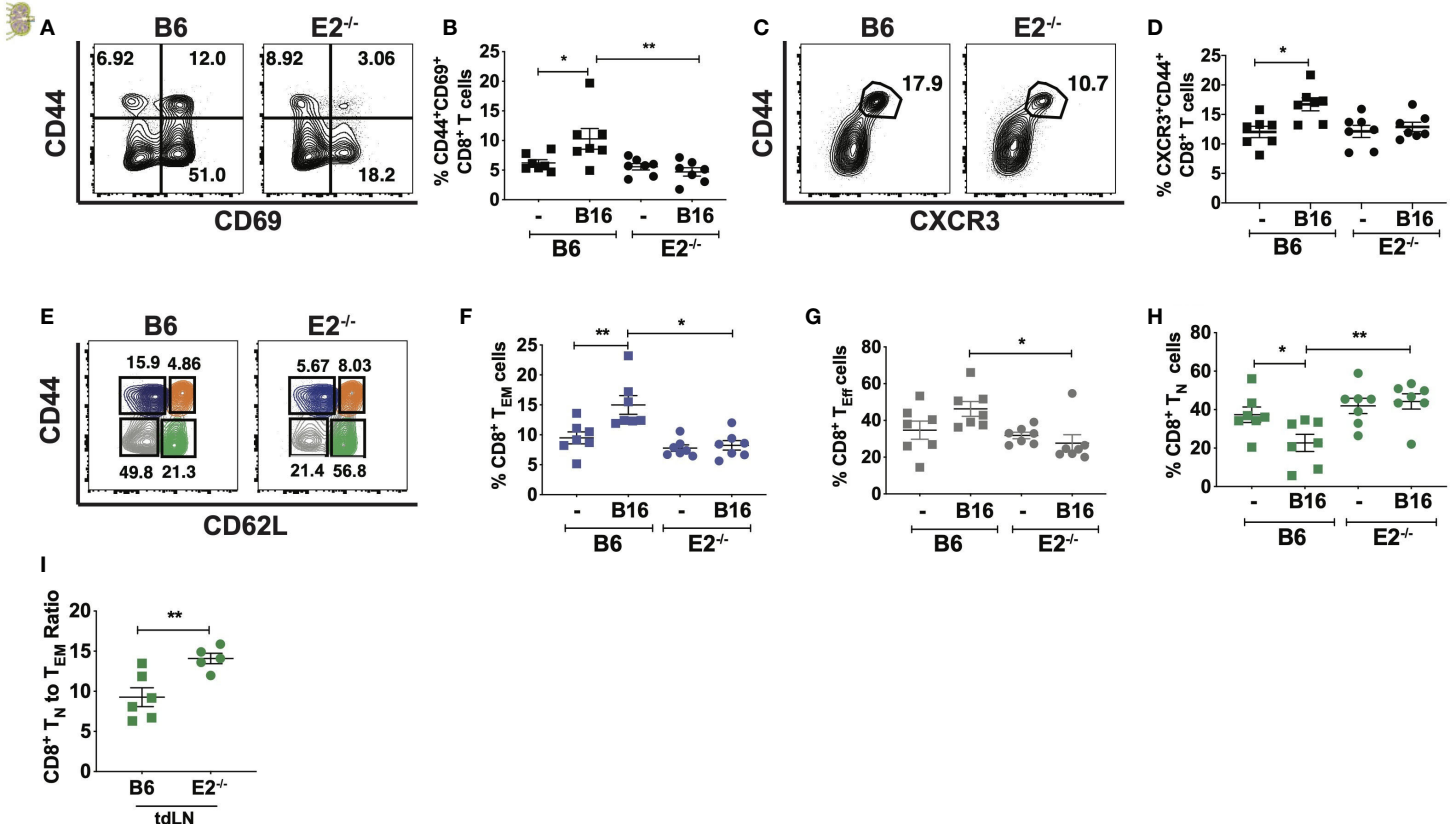
Loss of nonclassical monocytes with B16F10-OVA reduces CD8+ T cell activation and increases naive CD8+T cell frequency. Representative contour plots of CD69 by CD44 **(A)**, frequencies of CD44+CD69+CD8+ T cells **(B)** by TCRb+CD8+ T cells within B6 or E2^-/-^ lung draining mediastinal lymph nodes at 6 days post injection. Representative contour plots of CXCR3 by CD44 **(C)** within B6 and E2^-/-^ mediastinal lymph nodes **(D)** by TCRb+CD8+ T cells. Representative contour plots of CD62L by CD44 for CD8+TCRb+ T cells within B16F10-OVA-injected B6 and E2^-/-^ mice, detailing CD8+ T_N_ (green), T_CM_ (orange), T_EM_ (blue), and T_Eff_ (gray) gates **(E)**. CD8+ T_EM_ **(F)**, T_Eff_ **(G)**, and T_N_ **(H)** cell frequencies in B6 (n=7) and E2^-/-^ (n=7) medLNs. CD8+T_N_ to effector memory CD8+ T cell ratios **(I)** in tumor-draining inguinal lymph nodes (tdLNs) of B6 (n=5) and E2^-/-^ (n=5) mice implanted with B16-OVA for 19 days. Statistically significant differences were at *p < 0.05 and **p < 0.01 (One Way ANOVA **(B, D, F–H)** and unpaired t-test **(I)**. Results are expressed as mean + s.e.m. from 2 independent experiments.

To investigate the influence of nonclassical monocytes on T cell activation and memory formation during metastasis, C57BL/6 and E2^-/-^ mice were intravenously injected with B16F10-OVA melanoma cells, and T cell activation and memory responses were investigated in the lung-draining mediastinal lymph nodes via flow cytometry (gating strategy shown in **Supplemental Figure 2A**). At 6 days post-tumor cell injection, analysis of the draining medLNs revealed a 1.8-fold increase in the frequency of CD44^+^CD69^+^CD8^+^ T cells, a phenotype associated with early T cell activation, in B16F10-OVA tumor-bearing B6 mice (B16) compared to uninjected B6 mice (**Figures 2A, B**). Strikingly, there was no such increase in frequencies of CD69^+^CD44^+^CD8^+^ T cells in E2^-/-^ mice post-B16F10-OVA compared to uninjected control E2^-/-^ mice (**Figure 2B**). In addition to increased expression of the early T cell activation marker CD69, we observed a significant increase in effector-like CD8^+^CXCR3^+^CD44^+^ T cells within B6 mice compared to uninjected controls, but we again observed no significant enrichment within E2^-/-^ medLNs (**Figures 2C, D**).

As naïve T (T_N_) cells encounter antigen, they expand and differentiate into effector T cells (T_Eff_), endowed with the ability to release effector cytokines, and kill target cells by release of perforin and granzyme. Following expansion, a majority of these T_Eff_ cells die by apoptosis, but a fraction of them mature into long-lived memory cells, which are broadly divided into central memory (T_CM_) and effector memory (T_EM_) T cells (29–31). Because of the attenuated CD8^+^ T cell activation in E2^-/-^ medLNs with B16F10-OVA, we next investigated CD8^+^ naive and memory population frequencies (**Figures 2E–H** and **Supplemental Figure 2C**). Flow cytometry analysis using the markers CD44 and CD62L (**Figure 2E** and **Supplemental Figure 2B** for FMO controls) of CD8^+^ naive and memory populations in the lung-draining medLN at 6 days post B16F10-OVA inoculation revealed a 1.8-fold reduction in CD44^hi^CD62L^lo^ T_EM_ frequencies in the medLN of E2^-/-^ compared to B6 mice (**Figure 2F**). CD44^lo^CD62L^lo^ T_Eff_ -like cell frequencies were also reduced 1.7-fold (**Figure 2G**) in response to B16F10-OVA. Despite decrease CD8^+^ T_EM_ and T_Eff_ -like frequencies, CD8^+^ T_CM_ percentages remained unchanged (**Supplemental Figure 2C**). Concomitantly, E2^-/-^mediastinal draining lymph nodes displayed a 2-fold increase in CD44^lo^CD62L^hi^ T_N_ cell frequencies (**Figure 2H**). The ratio of CD8+T_N_ to T_EM_ cells was significantly higher in tumor-draining LN of E2^-/-^ mice (**Figure 2I** and **Supplemental Figure 2D**).

As a control measurement of tissue specificity, analysis of the inguinal lymph nodes revealed similar T_EM_ (**Supplemental Figure 2E**), T_N_ (**Supplemental Figure 2F**), and T_Eff_ -like (**Supplemental Figure 2G**) frequencies between genotypes at both baseline and post B16F10-OVA injection, indicating the occurrence of a select and specialized CD8^+^ T cell response in the lung-draining mediastinal lymph node in response to lung tumor growth. Finally, the B16F10-OVA-elicited T cell activation in the lung-draining LNs was specific to CD8^+^ T cells, as there was no detectable difference in activated CD4^+^ T cell or CD4^+^ T_EM_ frequencies within wild-type or E2^-/-^ tumor-draining mediastinal LNs (**Supplemental Figures 2H, I**). Thus, with nonclassical monocyte deficiency in the setting of lung cancer, there was selective reduction in CD8^+^ T cell activation and effector phenotype in tumor-draining lymph nodes in response to tumor that was not observed in CD4+ T cells.

### E2^-/-^ mice displayed reduced antigen-specific CD8 T cell responses

**Figure 3:**
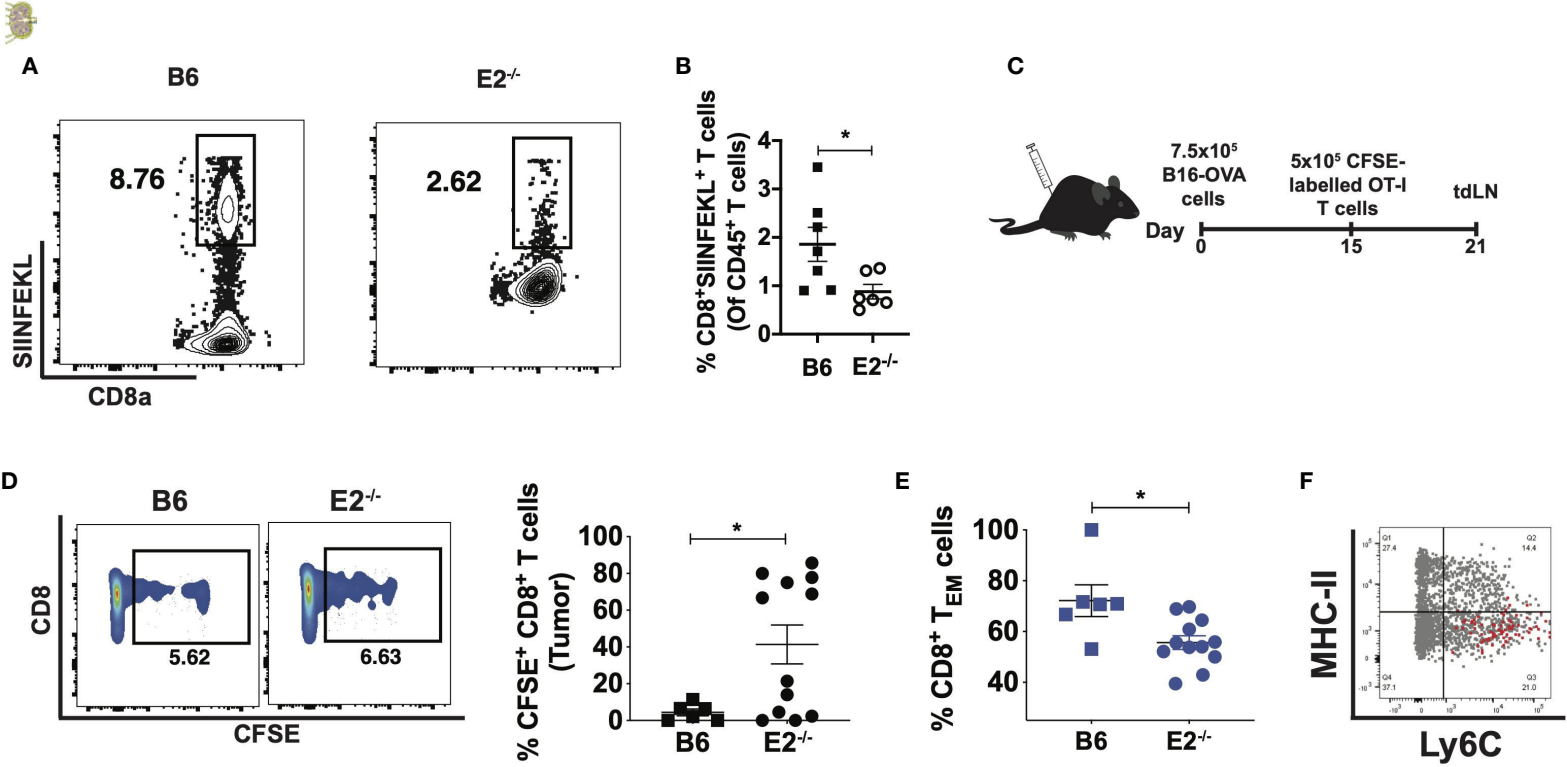
E2^-/-^ mice display attenuated antigen-specific CD8 T cell responses in the tumor and tumor-draining lymph nodes. Representative contour plots of CD8a by SIINFEKL, gated on live, singlet CD8a+TCRb+ T cells **(A)**. Pooled frequencies of CD8+SIINFEKL+ T cells of CD45+ cells within B6 (n=7) and E2^-/-^ (n=6) mice at 21 days post i.v. injection of B16F10-OVA **(B)**. Experimental schematic for assessing OT-I CD8+ T cell proliferation and memory formation in the tumor and tumor-draining lymph nodes **(C)**. CD8+CFSE+ percentages within tumors of B6 (n=6) and E2^-/-^ (n=12) mice from one experiment **(D)**, and pooled effector memory T cell frequencies within B6 (n=6) and E2^-/-^ tumors (n=5) **(E)**. Overlaid dot plot of MHC-II by Ly6C, gated on circulating CD11b+CD115+ monocytes (gray), with the MHC-I-SIINFEKL-peptide^+^ cells (red) **(F)**. Statistically significant differences were at *p < 0.02; (unpaired t-test).

To examine antigen-specific CD8^+^ T cell responses to tumors in the absence of nonclassical monocytes (**Figure 3**), we employed tetramer staining using SIINFEKL, a peptide recognized by CD8^+^ T cells that have been presented with the antigen in the context of I-A^b^. We observed a 2-fold reduction in SIINFEKL-specific CD8 T cells *in vivo* at 21 days following B16F10-OVA cell injection in E2^-/-^ mice (**Figures 3A, B**). We chose to examine these responses at 21 days after injection because our pilot studies (data not shown) indicated that antigen-specific T cells appear within the lymph nodes at this time point. These data suggest that nonclassical monocytes influence antigen-specific CD8+ T cell responses in tumor-draining lymph nodes *in vivo.*

### E2​-/- ​mice fail to elicit an effector memory CD8+​ ​T cell response after OT-I CD8​+​ T cell adoptive transfer

To further explore the impact of nonclassical monocyte deficiency on the mechanisms of CD8+​ ​T cell activation, we employed an OT-I adoptive transfer system. As OT-I CD8+​ ​T cells are specific for the OVA antigen, their adoptive transfer into animals injected with B16F10-OVA cells can reveal context-dependent defects in T cell activity within transgenic mouse models. B16F10-OVA cells were thus subcutaneously implanted into the flanks of B6 and E2^-/-^ mice and 15 days later splenic, CFSE-labeled OT-I CD8+​ ​T cells were injected directly into the tumor (**Figure 3C**). T cell activation and memory responses were assessed in tdLNs via flow cytometry 6 days post-injection (​**Figure 3C** and **Supplemental Figures 3A, B**​). Flow cytometric analysis revealed that transferred Vβ5+​ ​Vα2+​ ​T cells in E2^-/-^ mice contained a higher frequency of cells containing CFSE with little dilution (indicating less proliferation) compared to cells in B6 mouse tumors (​**Figure 3D**​), despite no significant difference in Vβ5+​ ​Vα2+​ ​frequencies (​**Supplemental Figure 3C**​). The viability of T cells was found to be similar (approximately 99%) for both B6 and E2^-/-^ T cells. Overall, these findings suggest that the E2^-​/-​^ tumor microenvironment prevents antigen-specific CD8+ T cells from proliferating. Analysis of CD62L and CD44 expression on the transferred OT-I CD8+​ ​T cells revealed a 1.3-fold reduction in CD8+​ T​EM​ cell frequencies (**Figure 3E**​). Thus, in the absence of nonclassical monocytes, transferred OT-I CD8^+^​ ​T cells were less effector memory-like in phenotype and less proliferative.

Multiple studies have shown that Ly6C^hi^ monocytes present antigens to activate T cells (21, 32–36). To investigate if Ly6C^lo^MHC-II^lo^ nonclassical monocytes are able to load tumor-specific antigen onto MHC-I complexes for cross-presentation to CD8^+^ T cells, levels of MHC-I-OVA-SIINFEKL^+^ cells present amongst circulating CD115^+^CD11b^+^ monocytes were quantified (**Figure 3F**). However, overlaid dot plots revealed that the MHC-I-SIINFEKL-peptide-expressing cells exclusively occupied the Ly6C^hi^MHC-II^lo^ classical monocyte compartment (**Figure 3F**), suggesting that nonclassical monocytes do not cross-present antigen, at least in the context of murine cancers.

### CD8^+^ T cells from E2^-/-^ mice display no defects in activation or cytokine production at homeostasis

**Figure 4:**
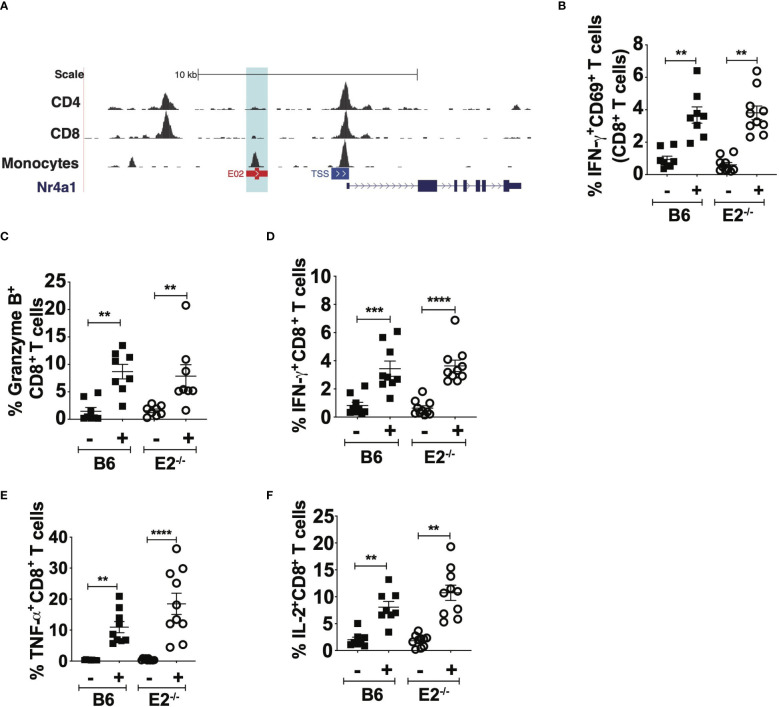
E2^-/-^ CD8+ T cells are not functionally impaired in pro-inflammatory cytokine production. BigWig Tracks of CD4 (GSM1463175), CD8 (GSM146174) to examine DNA accessibility at the E2 locus in primary CD4+ and CD8+ T cells, in addition to mouse monocytes **(A)**. Pooled frequencies of IFN-g+CD69+ **(B)**, Granzyme B+CD8+ **(C)**, IFNg+CD8+ **(D)**, TNFa+CD8+ **(E)**, and IL-2+CD8+ **(F)** T cells. CD8+ T cells were purified via negative selection from the spleens of B16F10-OVA injected B6 (n=7) and E2^-/-^ (n=7) mice and stimulated with aCD3, CD28 for 48hrs, followed by PMA/Ionomycin with Brefeldin A for 5hrs. Results are expressed as mean + s.e.m. for two independent experiments. Statistically significant differences were at **p < 0.01, ***p < 0.001, ****p < 0.0001; (One-Way ANOVA).

Nr4a1, the transcription factor downstream of the E2 enhancer that controls Ly6C^lo^ monocyte development (18), is also expressed by T cells and is critical for their differentiation and egress from the thymus (37, 38). Therefore, we asked if our observed attenuated CD8^+^ T cell antigen-specific responses in E2^-/-^ mice were possibly due to deletion of the E2 enhancer in T cells. We visualized the E2 enhancer region on T cell subsets utilizing previously-published ATAC seq datasets (39). Analysis of the E2 enhancer region between CD4^+^ T cells, CD8^+^ T cells, and monocytes revealed an open chromatin pattern in monocytes, but not in CD4^+^ and CD8^+^ T cells (light blue box in **Figure 4A**). Conversely, robust ATAC peaks were observed at the TSS of the *Nr4a1* locus in all cell types (**Figure 4A**). Thus, deletion of the E2 enhancer is unlikely to interfere with *Nr4a1* gene transcription in T cells. However, to confirm that deficiency of the E2 enhancer did not cause intrinsic functional changes in CD8^+^ T cell responses, B6 and E2^-/-^ CD8^+^ T cells were purified from spleens and lymph nodes at 6 days post injection of B16F10-OVA and stimulated with anti-CD3/CD28. Polyclonal stimulation of B6 and E2^-/-^ CD8^+^ T cells for 48 hours elicited similar frequencies of pro-inflammatory IFNγ^+^CD69^+^CD8^+^ T cells (**Figure 4B**). Frequencies of CD8^+^ T cells positive for granzyme-B (**Figure 4C**), IFNγ (**Figure 4D**), TNFα (**Figure 4E**), or the T cell growth factor IL-2 (**Figure 4F**) were similar in B6 and E2^-/-^mice. These results collectively indicate that E2^-/-^ CD8^+^ T cells are equally responsive to polyclonal stimulation and that no inherent deficiency in T cell responses is introduced with deletion of the E2 enhancer region *in vivo*.

### Nonclassical monocytes migrate to the lungs, then lymph nodes in response to lung tumors

**Figure 5:**
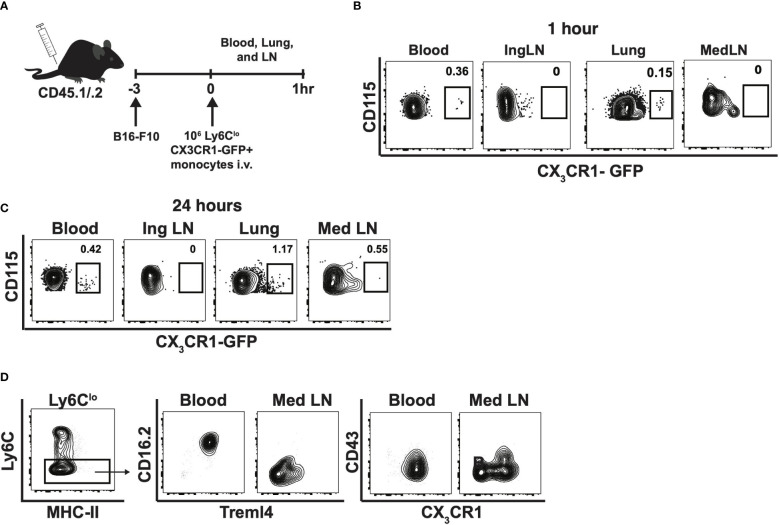
Nonclassical monocytes traffic to lung, then lymph nodes in response to tumor and change phenotype in the tumor-draining lymph nodes. Experimental schematic of adoptive transfer into CD45.1/2 mice **(A)**, and contour plots of CX3CR1-GFP by CD115 within blood, inguinal lymph node (Ing LN), lung, and medLN at 1h **(B)** and 24h. Data are from an individual mouse **(C)**. Representative contour plots of Treml4 by CD16.2 and CX3CR1 by CD43 within the blood and mediastinal lymph nodes of B6 mice. Representative of two experiments **(D)**.

To determine whether Ly6C^lo^ monocytes preferentially homed to the medLNs in the context of cancer, 1x10^6^ sorted nonclassical monocytes expressing CX3CR1-GFP were injected into B16F10 inoculated CD45.1/.2 mice (**Figure 5A**). Subsequently, as early as 1-hour post injection, CX_3_CR1-GFP^+^CD115^+^ monocytes were readily detectable within the blood and lungs, but were absent from mediastinal LNs (**Figure 5B**). At 24h post-injection, CX_3_CR1-GFP^+^CD115^+^ monocytes were detected in the lungs and in the tumor-draining mediastinal LNs, but not in the control inguinal LNs (**Figure 5C**). These results suggest that Ly6C^lo^ monocytes preferentially traffic to the lungs, rather than to the medLNs, during early tumor seeding in mice. Interestingly, Ly6C^lo^ monocytes within the lung-draining medLNs were phenotypically distinct from circulating nonclassical monocytes in that they displayed decreased CD16.2, Treml4, and CX_3_CR1 expression (**Figure 5D**), suggesting that alterations in monocyte phenotype occur within these tumor-draining lymph nodes.

### E2^-/-^ mice display decreased CD8^+^ T cell maturation in a model of lung seeding

**Figure 6:**
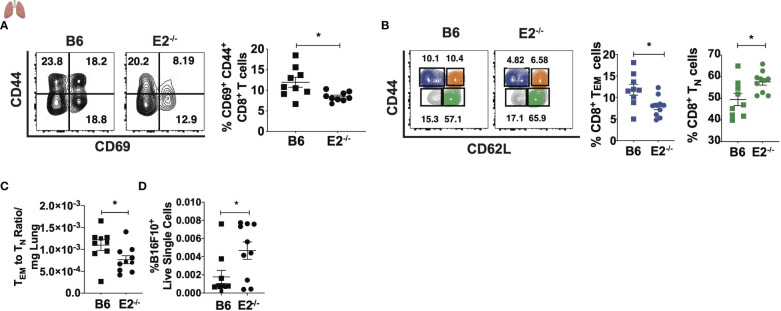
Attenuated CD8^+^ TEM cell frequencies in lungs of E2^-/-^ mice compared to B6 mice. Representative contour plots of CD44 by CD69 **(A)** and pooled CD69+CD44+ frequencies of B6 and E2^-/-^ mice, gated on CD45+CD8+TCRb+ T cells. Representative contour plots of CD62L by CD44, gated on CD45+CD8+TCRb+ T cells **(B)**. CD8+ TEM **(B)**, CD8+ TN cell frequencies **(B)**, TEM to TN ratio/mg lung **(C)**, and frequencies of Zs-Green-B16F10+ cells in lung at day 6 post injection **(D)**. Results are expressed as mean + s.e.m. from one of two independent experiments by B6 (n=9) and E2^-/-^ (n=10) mice. Statistically significant differences were at *p < 0.05 (unpaired student’s t-test).

As E2^-/-^ mice display decreased mature T cell responses during metastasis in the lung-draining medLNs, we investigated whether T cells within the lung metastatic environment were altered in the absence of nonclassical monocytes. Similar to the phenotype in the medLNs, lungs from E2^-/-^ mice had fewer activated CD8^+^ T cells (**Figure 6A**) compared to the lungs of B6 mice. Upon examining frequencies of T_N_ and T_EM_ cells in the lung, we found a shift in the CD8^+^ T cell population to a more naïve-like phenotype (**Figure 6B**), similar to what we have observed in tumor-draining LNs (**Figure 2**). We observed a significant reduction in the ratio of T_EM_ to T_N_ cells within E2^-/-^ lungs compared to B6 lungs (**Figure 6C**). Thus, in addition to medLNs, CD8^+^ T_EM_ responses were similarly attenuated in the lungs of E2^-/-^ mice. Corroborating this increased T_N_-like profile in the lung was a 2.6-fold increase in B16F10 cancer particles in the lungs of E2^-/-^ compared to B6 mice at 6 days post injection (**Figure 6D**). These data suggest that an altered tumor microenvironment with nonclassical monocyte deficiency promotes T cell skewing away from a T_EM_ phenotype in the lung and lung-draining lymph nodes, likely contributing to increased tumor cell seeding in early metastasis.

### The chemokine CCL21 is reduced in lungs with nonclassical monocyte deficiency

**Figure 7:**
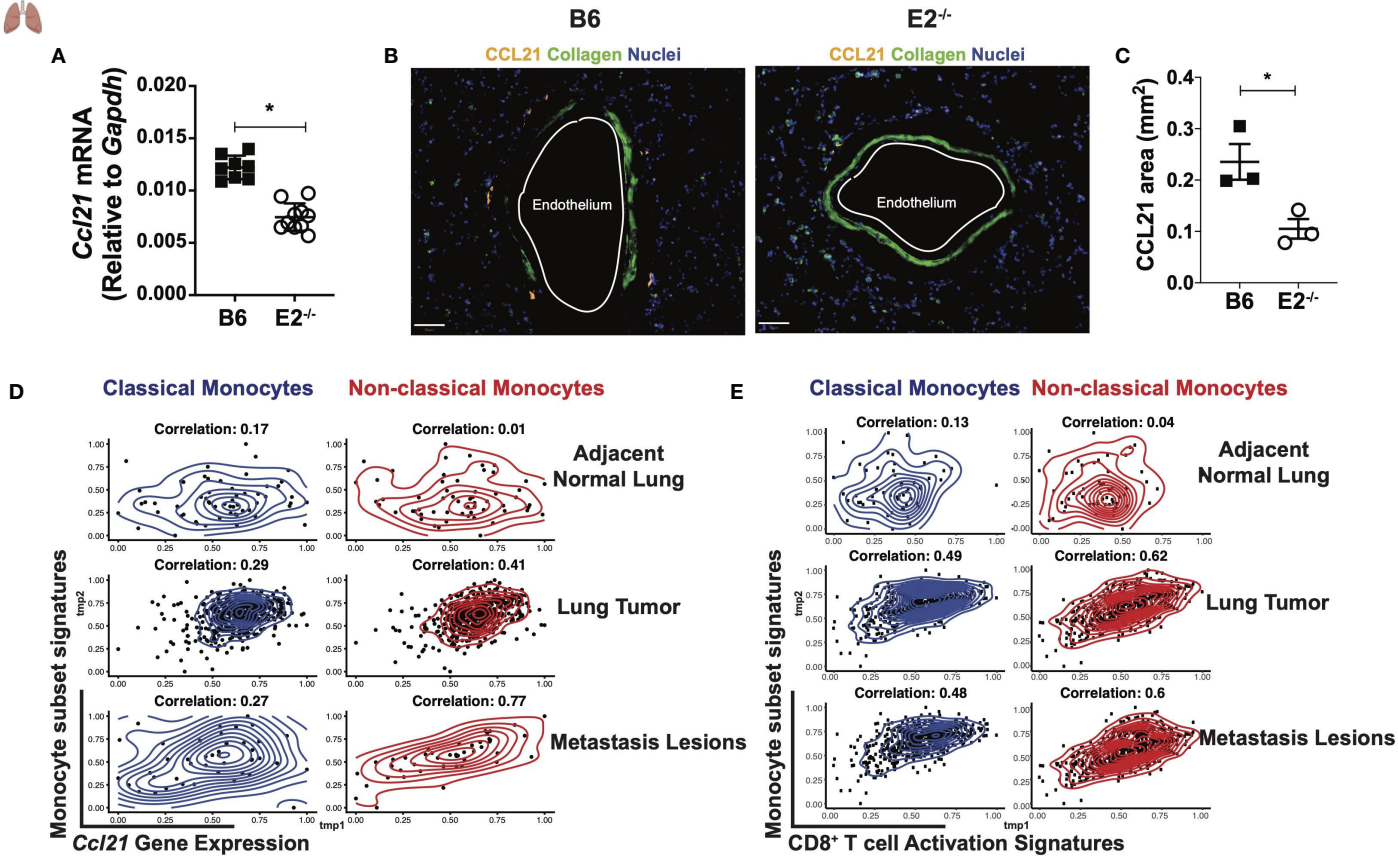
Altered tumor micro-environment in E2^-/-^ mice lacking non-classical monocytes. *Ccl21* mRNA abundance within B6 and E2^-/-^ lungs at homeostasis **(A)**. Representative lung sections stained for CCL21, Collagen, and Nuclei within lungs of B6 (n=3) and E2^-/-^ (n=3) mice at 6 days post injection with B16F10-OVA. **(B)**. Pooled CCL21 area for B6 (n=3) and E2^-/-^ (n=3) mice at 6 days post B16F10-OVA injection **(C)**. Results are expressed as mean + s.e.m. from two experiments **(A)** and one experiment using multiple mice and processed at the same time to avoid batch issues **(B, C)**. Statistically significant differences were at *p < 0.05 (unpaired student’s t-test). Spearman correlation of *Ccl21* **(D)** and CD8+ T cell activation signatures **(E)** with classical or non-classical monocyte gene signatures in human subjects with either melanoma (n=32) from (40) or adenocarcinoma (n=230) from the (41).

We reasoned that since fewer mature T cells are present at early time points during metastasis, that perhaps the lung environment is not sufficiently primed for an anti-tumor response in the absence of nonclassical monocytes. A crucial chemoattractant for recruitment of T cells to peripheral tissues is the chemokine CCL21, which binds to its receptor CCR7, typically expressed on T_N_ cells. Prior to injection of B16F10 cancer cells, lungs from E2^-/-^ mice initially expressed significantly less CCL21 transcript compared to B6 mice (**Figure 7A**). We also found a stark reduction of CCL21 in the blood vessels of normal adjacent lung tissue in E2^-/-^ mice that were analyzed 6 days after B16F10 injection (**Figure 7B**). The endothelium was identified by CD31 staining and morphology (data not shown). In the absence of nonclassical monocytes, we observed a 2.3-fold reduction in the CCL21-positive area (mm^2^) within the lungs (**Figure 7C**), suggesting that nonclassical monocytes are necessary for CCL21-mediated early recruitment of T cells to the tissue for an effective anti-tumor response. In addition to attracting T_N_ cells, CCL21 elicits T cell activation and expansion and a pro-inflammatory Th1 profile. Importantly, CCL21 expression within established tumors correlates with increased pro-inflammatory T cell infiltration and heighted anti-tumorigenic responses (42–45). We next analyzed published datasets from 38 human subjects with melanoma (40) and 230 human subjects with adenocarcinoma (41). We found a positive correlation with *Ccl21* transcript levels and the presence of nonclassical monocytes in human metastatic lesions and primary lung tumors, when compared to adjacent normal lung tissue in these 2 cohorts (**Figure 7D**). These relationships were much stronger for nonclassical (red) than classical (blue) monocytes (**Figure 7D**). Thus, nonclassical monocytes influence CD8^+^ T_EM_ cell maturation, via the chemokine CCL21, that are responsible for blocking tumor seeding in the lungs.

### Genes linked to CD8^+^ T cell activation correlate with nonclassical monocytes in lung cancer

To compare the relationship between classical and nonclassical monocytes with CD8^+^ T cell activation in the context of human lung cancer, Spearman correlations were performed to evaluate the association between CD8+ T cell activation genes and gene signatures of classical and nonclassical monocytes in adjacent normal lung, lung tumor, and metastatic melanoma lesions utilizing the same publicly available datasets mentioned above (40, 41). One correlation analysis was performed for all possible pairs of signatures in each tissue. Interestingly, nonclassical monocyte signatures correlated more strongly with CD8^+^ T cell activation in lung tumor and metastasis lesions than did classical monocytes (**Figure 7E**). These data support our findings that Ly6C^lo^ monocytes selectively modulate CD8^+^ T cell activation in the lung and tumor-draining lymph nodes. Thus, nonclassical monocytes may represent an attractive target for increasing CD8^+^ T_EM_ responses in lung cancers.

## Discussion

Our laboratory previously demonstrated that Ly6C^lo^ nonclassical monocytes orchestrate the killing of metastatic tumor cells in the lungs (25, 26). However, whether the nonclassical monocytes influenced T cell functions was not tested in our earlier work. The production of an adequate CD8^+^ T_EM_ response is critical for generating potent anti-tumor immunity. For example, increased intratumoral CD8^+^ T_EM_ cells observed in patients on α-PD-1 blockade correlate with improved responses to anti-cancer treatment (46). Reduced access to the homeostatic cytokines IL-7 and IL-15, critical for memory CD8^+^ T cell survival and maintenance, negatively impaired anti-tumor effector CD8^+^ T cell responses (47). Thus, the acquisition of T_EM_ responses are essential for potent anti-tumorigenic T cell immune responses. Within this current study, we found that in the absence of nonclassical monocytes, CD8^+^ T_EM_ responses to early metastatic cancer cell seeding were severely attenuated in lung and in tumor-draining lymph nodes. This reduction in robust T cell responses were not observed in CD4+ T cells.

In this study, we utilized E2-deficient mice, which are lacking a 1500bp enhancer region upstream of the transcriptional start site of *Nr4a1*. These mice lack nonclassical, patrolling monocytes, as confirmed here and previously by our group (26). We have previously reported that macrophage inflammatory responses are normal in these mice (26), and we show here in this study that T cell responses are normal. However, it is possible that other innate immune cells, including neutrophils, are changed in frequency in the E2-/- mice. Studies to examine neutrophil frequencies and heterogeneity in these mice are ongoing.

A seminal study from a few years ago demonstrated that Ly6C^hi^ monocytes enter steady-state non-lymphoid organs and recirculate to lymph nodes without differentiating into macrophages or DCs (35). In our current study, we found that the frequency of CD115^+^ monocytes in the lungs draining lymph nodes increased after B16F10-OVA administration. Moreover, Ly6C^lo^ nonclassical monocytes preferentially trafficked to the lungs after injections of B16F10-OVA, but then appeared in tumor-draining mediastinal lymph nodes. However, we did not examine whether these monocytes extravasated into lung tissue itself, or simply moved through the lung vasculature prior to entering the lymph nodes. Phenotyping of the myeloid compartment revealed that monocytes were one of the most prevalent immune cells in the lungs. The frequency of monocytes in the LNs was lower than macrophage frequencies, but similar to DC frequencies.

In contrast to Ly6C^lo^ monocytes, studies have demonstrated that Ly6C^hi^ monocytes migrate into the LN and present antigen to T cells (21, 32–36). We observed that nonclassical Ly6C^lo^ monocytes in the tumor-draining mediastinal lymph nodes were phenotypically distinct from Ly6C^lo^ monocytes in the blood, displaying lower expression of Treml4 and CD16.2. Treml4, a protein implicated in cross presentation and antigen uptake, is expressed on monocytes, CD8α^+^ dendritic cells, and macrophages (48). This complements our finding that Ly6C^lo^MHC-II^lo^ monocytes do not express MHC-I. Additionally, another study utilizing transcriptional profiling of blood, lung, and LN monocytes revealed a very close association compared to LN resident or migratory DCs (35). Nevertheless, these studies demonstrate that Ly6C^lo^MHC-II^lo^ monocytes within different compartments are phenotypically distinct. Future studies will interrogate the transcription factors that distinguish monocytes between the lungs, LNs, and periphery.

CCL2 and CCL7 have been previously implicated in recruitment of Ly6C^hi^ monocytes (49). We observed a significant reduction in the chemokine CCL21, also referred to as secondary lymphoid tissue chemokine (SLC) and the ligand for CCR7-expressing cells (50), within E2^-/-^ compared to control B6 lungs, both at homeostasis and after tumor seeding. Originally identified within lymphoid tissues, CCL21 facilitates the colocalization of naive T cells with DCs in the T cell zone, ensuring cell-cell contact and T cell activation (51). In addition to lymphoid tissue, CCL21 is also highly expressed in non-lymphoid tissues, such as the lung (52). CCL21 is required for T cell migration to the lung, as analysis of T cells within mice receiving α-CC21 treatment revealed a robust diminution in CD4^+^ and CD8^+^ T cell numbers (52). While naive T cells depend on CCL21 for their recruitment to secondary lymphoid organs, the trafficking of memory T cells was found to be dependent on CCL21 in autoimmunity (53). Extensive work in cancer immunotherapy has shown that the CCL21/CCR7 expression axis promotes growth and metastasis in a variety of tumor types, including melanoma, breast, thyroid, colon, head, and neck cancers (54–59). Knockdown of *Ccl21* gene expression also severely attenuates metastatic tumor growth. These studies implicate CCL21 as an important target in cancer immunotherapy.

We hypothesize that the Ly6C^lo^ nonclassical monocytes that patrol the vasculature elicit CCL21 production by endothelial cells, which in turn stimulates T cell recruitment to lung and lung-draining lymph nodes in response to cancer. However, we did not examine the lymphatic vasculature in this study, nor did we closely examine vascular endothelial function. We cannot rule out that there are additional changes in endothelial homeostasis within nonclassical monocyte-deficient in E2^-/-^ mice. We have previously shown that mice with monocyte-specific loss of kindlin-3, in which nonclassical monocytes are present but have defective and inefficient patrolling of the vasculature, display an inflamed endothelium (60). Thus, future studies will need to focus on understanding how Ly6C^lo^ monocytes modulate CCL21 production and other endothelial functions in the setting of cancer.

In summary, our study demonstrates that nonclassical monocytes are important for the generation of a potent CD8^+^ T_EM_ phenotype in the lungs and lymph nodes during early metastasis. Movement away from this CD8^+^ T_EM_ phenotype towards an T_N_ phenotype is responsible, at least in part, for the increased lung metastasis observed in nonclassical monocyte-deficient E2^-/-^ mice. Collectively, our work identifies a previously undiscovered role for Ly6C^lo^ monocytes in modulating CD8^+^ T cell activation/memory responses via CCL21.

## Materials and methods

### Mice

C57BL/6 (000664), CD45.1/.2, C57BL/6-Tg(TcraTcrb)1100Mjb/J (003831, OT-I), MacGreen, and CX3CR1-GFP (B6.129P2(Cg)-*Cx3r1^tm1Lit^* ^t^/J) were purchased from the Jackson Laboratory, while E2-deficient (E2^-/-^) mice (26) were bred and maintained in the La Jolla Institute for Immunology (LJI) animal facility. Mice were fed a standard rodent chow diet and housed in microisolator cages in a special pathogen free facility. All experiments followed the guidelines of the La Jolla Institute for Immunology (LJI) Animal Care and Use Committee, and approval for use of rodents was obtained from LJI according to criteria outlined in the Guide for the Care and Use of Laboratory Animals from the National Institutes of Health. Mice were euthanized by CO_2_ inhalation following approved guidelines. The B16F10-OVA melanoma cell line was obtained from ATCC.

### Flow cytometry

Immediately following euthanasia, cardiac blood draw and transcardial perfusion of PBS with EDTA was performed. Lungs and tumor-draining lymph nodes (mediastinal or inguinal) were explanted. Lungs were lavaged with Dulbecco’s PBS (DPBS) (Gibco) containing 2 mM EDTA, excised, and mechanically digested over a 70uM cell strainer for T cell analysis.

For macrophage/DC quantification, lungs were digested for 45 minutes at 37°C using 0.26 U ml^−1^ Liberase™ (Roche) and 0.25 mg ml^−1^ DNaseI (Roche) in 5ml DMEM. Samples were placed in C-Tubes (Miltenyi) and briefly processed with a GentleMACS Dissociator (Miltenyi). Samples were then incubated at 37°C for 30 min and processed a second time via GentleMACS. Tissue homogenate was then passed through a 70uM filter.

T cell frequencies and phenotypes within lymph nodes and lungs were assessed by staining with anti-mouse CD8a (53-6.7), CD44 (IM7), CD45 (30-F11), CD62L (MEL-14), CD69 (H1.2F3), TCRβ (H57-597), CXCR3 (CXCR3-173), Vβ2 (B20.6), and Vβ5.1, 5.2 (MR9-4). Cytokine production by purified CD8^+^ T cells was assessed by staining with anti-mouse Granzyme B (GB11), IFNγ (XMG1.2), IL-2 (JES6-5H4) (BD Biosciences), and TNFα (MP6-XT22). Monocyte, macrophage, and DC frequencies were assessed by staining with the following antibodies: CD8a (53-6.7), CD11b (M1/70), CD11c (N418), CD16.2 (FcγRIV) (9E9), CD43 (S11), CD64 (X54-5/7.1), CD103 (2E7), CD115 (AFS98), I-A^b^ (AF6-120.1), H-2K^b^ bound to SIINFEKL (25-D1.16), Ly6C (HK1.4), Treml4 (I6E5). All antibodies were used at a 1:100 dilution except for CD44 (1:400), CD45 (1:300), and CD19 (1:200). Live/Dead stain (at a 1:800 dilution of Live/Dead Fixable Yellow from Invitrogen) was used in all experiments. Cells were acquired on the LSR-Fortessa (San Diego, CA).

### Tetramer staining

The MHC Class I peptide tetramer, SIINFEKL/K^b^ was synthesized by the NIH tetramer core facility (Emory, GA). LN single cell suspensions were incubated with PE-conjugated SIINFEKL/K^b^ on ice, followed by surface antibodies at 4°C for 30’.

### OT-I CD8​+ T​ cell adoptive transfer

C57BL/6 and E2-​/-​ mice were implanted with 1.75x10^5^ B16F10-OVA cells in Matrigel. CD8+​ ​T cells were purified from OT-I mice via negative selection using a CD8+​ ​T cell isolation kit (StemCell) and labelled with 1μM CFSE (Invitrogen). Purity was routinely greater than 90%. Five hundred thousand isolated CD8+​ ​T cells were transferred intratumorally into tumor-bearing C57BL/6 or E2^-​/-^ ​recipients at 15 days post implantation. T cell activation and proliferation within tumors and tumor draining lymph nodes (tdLN) were assessed at six days post implantation via flow cytometry.

### Ly6C^lo^ adoptive transfer

Ly6C^lo^ (CX3CR1^hi^) monocytes were sorted from blood of CX3CR1-GFP mice and 10^5^ Ly6C^lo^ monocytes were transferred i.v. into CD45.1/.2 recipients. At 4hrs and 24hrs post transfer, the frequency of Ly6C^lo^ monocytes was assessed in blood, inguinal lymph nodes (IngLN), lung, and mediastinal lymph nodes (MedLN) via flow cytometry.

### Imaging

The presence of CD115^+/gfp^ monocytes in the mediastinal lymph node (LN) was assessed by confocal imaging using a Zeiss LSM 880 with Airyscan after agarose embedding and vibratome sectioning. LNs were dissected from MacGreen mice 8 hours post B16F10-RFP injection and fixed for 2 hours in 2% PFA freshly prepared at room temp, followed by embedding in 3% w/v agarose. Samples were sectioned at 100 µm thickness using a Leica Biosystems vibratome and mounted in PBS for imaging the following day.

B16F10 melanoma cells expressing luciferase were injected IV (3×10^5^ per mouse) through the tail vein. Luciferase activity was measured using an IVIS 200 Bioluminescence Imager (Caliper Life Sciences) after IV injection of 1 mg D-Luciferin (Caliper Life Sciences) in 100 μL PBS.

### Quantitative real time PCR

Total cellular RNA from B6 and E2^-/-^ lungs was extracted by Trizol (Life Technologies) followed by RNA purification using Direct-zol™ RNA miniPrep (Zymo Research, Irvine, CA) per manufacturer’s instructions. RNA purity and quantity was determined by nanodrop spectrophotometer (Thermo Scientific), and equal amounts of RNA were used to synthesize cDNA using an iscript cDNA synthesis kit (Bio Rad). mRNA expression was measured in real time quantitative PCR using Taqman gene expression system and *Gapdh* (Mm99999915_g1) and *Ccl21* (Mm03646971_gH) primers (Applied Biosystems). Relative gene expression levels were calculated using the 2^-ΔΔCT^ method. *Gapdh* was set to 1.

### CCL21 analysis within the lung TME

Lungs of B6 and E2^-/-^ mice injected with B16F10-OVA were sectioned and analyzed for CCL21. Lungs were cardiac perfused first with 10 mL PBS containing 2 mM EDTA until the lungs turned white and inflated, followed by an additional 10 mL of PBS containing 2% PFA freshly prepared. Lungs were then removed and fixed by immersion in additional PBS containing 2% PFA at 4°C for 4 hours. Samples were sucrose exchanged overnight by first washing in PBS 3x, then immersing in PBS with 30% w/v sucrose with 0.05% NaN_3_. Finally, lungs were frozen in OCT and cryo-sectioned at 20 µm. Staining was performed using Sequenza racks and CCL21 antibody (R&D Systems AF457) overnight at 4C, and secondary donkey anti-goat Northern Lights 557 conjugated antibody (R&D Systems NL001) for 2 hours at room temp. Nuclei were stained using Hoechst 33352 (Thermo Fisher H3570) for 30 minutes at room temp before mounting in Prolong Gold (Thermo Fisher P36930). Whole lung images were obtained using a Zeiss LSM 880 with Airyscan. QuPath software was used for analysis of the images to quantify CCL21 area per total area of lung (61). A pixel classifier was trained on one B6 and one E2^-/-^ lung sample, then applied to batch processing for the remaining 4 samples (2 B6 and 2 E2^-/-^) using the same parameters.

### Correlation of CD8^+^ T cell activation signatures and Ccl21 with classical/nonclassical monocytes

Spearman correlation of gene signatures of nonclassical monocytes (LAIR2, ASAH1, APOBEC3A, TSPAN14, LIPA, CYTIP, SIGLEC10, LILRB1) and classical monocytes (CD14, VCAN, S100A8, S100A9, FCN1, ITGB2, LRP1, CSF3R) (62) with CD8^+^ T cell activation gene signatures (T-bet, Podoplanin, CCR7, CXCR5, IL-12a, IL-12b, IL-7) in TCGA lung cancer (tumor and adjacent normal samples (41)) and in metastasis lesion samples from melanoma patients (40).

### Statistical analysis

All statistical analyses were performed in Graphpad Prism v. 8.0. Error bars represent the mean ± s.e.m., as indicated by the N listed in each figure legend. Results were analyzed by either unpaired two-tailed student’s t-test, one-way analysis of variance (one way ANOVA), or two-way ANOVA followed by Tukey’s multiple comparisons test. Pearson correlation coefficient (*r*) was used to determine the statistical significance and correlation with human studies. A *p*-value of 0.05 was considered as significant.

## Data availability statement

The original contributions presented in the study are included in the article/**Supplementary Material**. Further inquiries can be directed to the corresponding author.

## Ethics statement

The animal study was reviewed and approved by La Jolla Institute for Immunology.

## Author contributions

LP, PM, CO, and CH performed experiments, analyzed data, and prepared figures. DA and AA analyzed data, prepared figures, and edited the manuscript. LP, PM, CO, and CH conceptualized the project; LP, PM, and CO wrote the manuscript. HQD performed correlations with publicly available data-sets. KS and MM performed experiments. WK performed CD115 confocal microscopy studies. GT generated the E2^-/-^ mouse strain in the Hedrick laboratory. CH was involved in experimental design, data analyses, and writing of the manuscript. All authors contributed to the article and approved the submitted version.

## Supplementary material

Supplementary Figure 1 Gating strategies used in this paper. Myeloid cell gating strategy within B6 and E2^-/-^ lungs **(A)**. Frequencies of CD4^+^TCRb^+^ and CD8^+^TCRb^+^ T cells within lung and lung-draining mediastinal lymph nodes of B6 and E2-def mice six days post-injection with B16 **(B)**. Myeloid cell gating strategy within mediastinal lymph nodes of B6 and E2-def mice **(C)**. The lineage (dump) gate for myeloid cells included CD3 (T cells), CD19 (B cells), NK1.1 (NK cells), and Ly6G (neutrophils).

Supplementary Figure 2 Gating strategy for assessing CD8+ T cell activation/memory responses in the mediastinal LN and lung. CD4+TCRb+ and CD8+TCRb+ T cells were gated by lymphocytes, followed by sequential single cell gates to remove doublets (FCS-A by FSC-H; SSC-A by SSC-H), live cells (Live-dead by SSC-A), CD45+ leukocytes (CD45 by SSC-A) **(A)**. Naive, central memory, effector memory, and effector T cells were gated utilizing fluorescence minus one (FMO) controls for CD44 and CD62L, gated on CD8+TCRb+ T cells **(B)**. Pooled central memory CD8+ T cells frequencies in the mediastinal lymph nodes of B6 and E2^-/-^ mice at 6 days post i.v. injection of B16F10-OVA **(C)**. Numbers of CD8+ TEM cells in mediastinal tumor-draining LNs in B6 and E2^-/-^ mice **(D)**. Pooled frequencies of CD8+ effector memory **(E)**, naive **(F)**, and effector **(G)** T cells in the inguinal lymph nodes at 6 days post i.v. injection of B16F10-OVA. Pooled frequencies of CD44+CD69+CD4+ T cells, gated on live, CD45+CD4+TCRb+ T cells in the mediastinal lymph nodes of B6 and E2^-/-^ mice at 6 days post i.v. injection of B16F10-OVA **(H)**. Pooled frequencies of CD4+ effector memory T cells at 6 days post i.v. injection with B16F10-OVA **(I)**. Results are expressed as mean + s.e.m. from two independent experiments. CD8 T_EM_ numbers and CD8 Teff numbers in D were quantified by an unpaired t-test. C and E-I were quantified by One-way ANOVA.

Supplementary Figure 3 Gating strategy for detecting adoptively-transferred OT-I CD8+ T cells. Transferred OT-I CFSE+ T cells were gated utilizing a no transfer control **(A)**. Pooled Vb5+Va2+ T cell frequencies, gated on CD8+ T cells in B6 (n=6) and E2^-/-^ (n=12) mice. Results are expressed as mean +/- SEM from one of three independent experiments (unpaired student’s t-test).
