# Supplementary figures and images for: Nonclassical monocytes potentiate anti-tumoral CD8+ T cell responses in the lungs
Source: Front Immunol. 2023 Jun 22;14:1101497. doi: 10.3389/fimmu.2023.1101497 (PMC10325638; doi:10.3389/fimmu.2023.1101497)

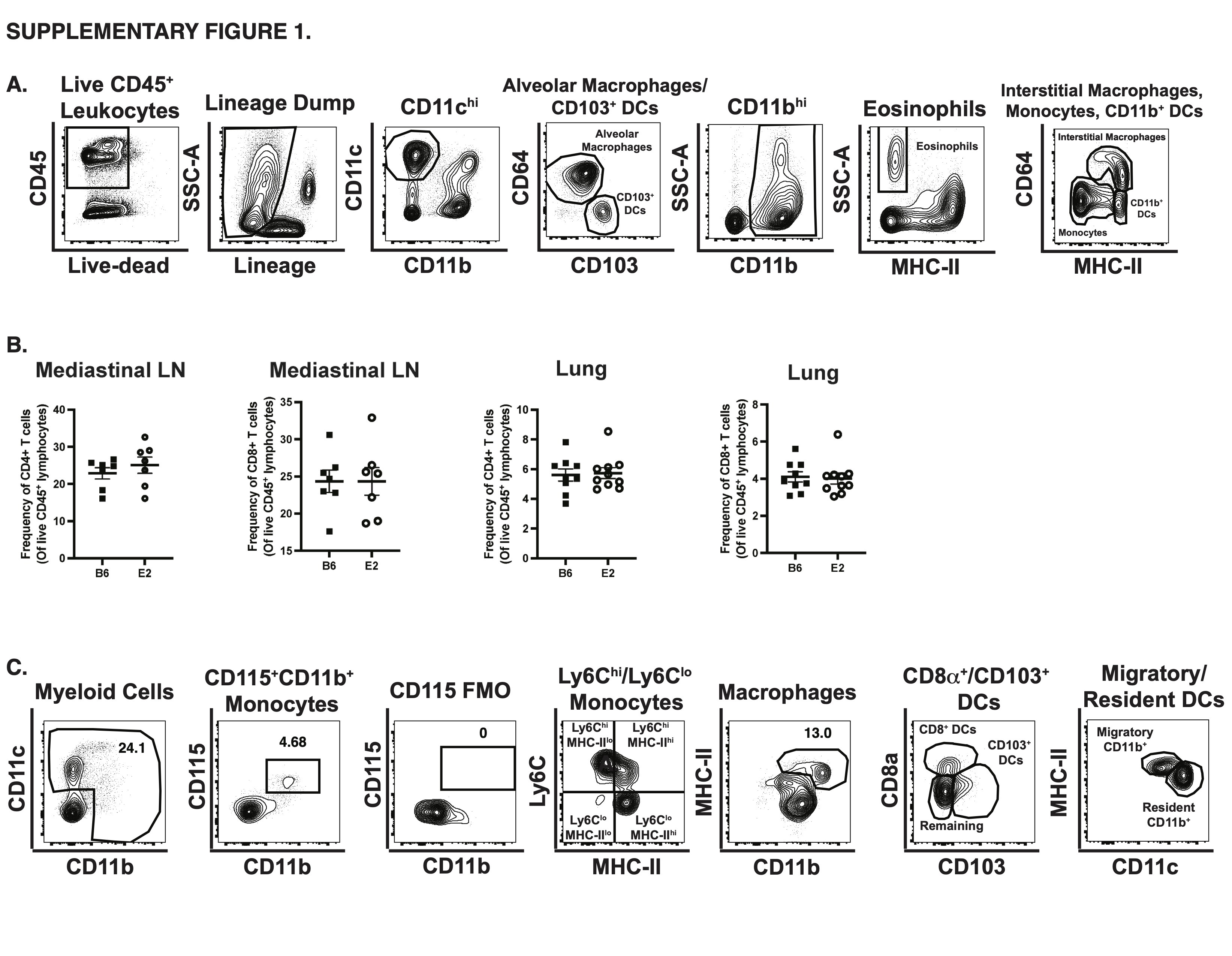

Supplement: Supplementary Figure 1 — Gating strategies used in this paper. Myeloid cell gating strategy within B6 and E2-/- lungs (A). Frequencies of CD4+TCRb+ and CD8+TCRb+ T cells within lung and lung-draining mediastinal lymph nodes of B6 and E2-def mice six days post-injection with B16 (B). Myeloid cell gating strategy within mediastinal lymph nodes of B6 and E2-def mice (C). The lineage (dump) gate for myeloid cells included CD3 (T cells), CD19 (B cells), NK1.1 (NK cells), and Ly6G (neutrophils). [file Image_1.jpeg]

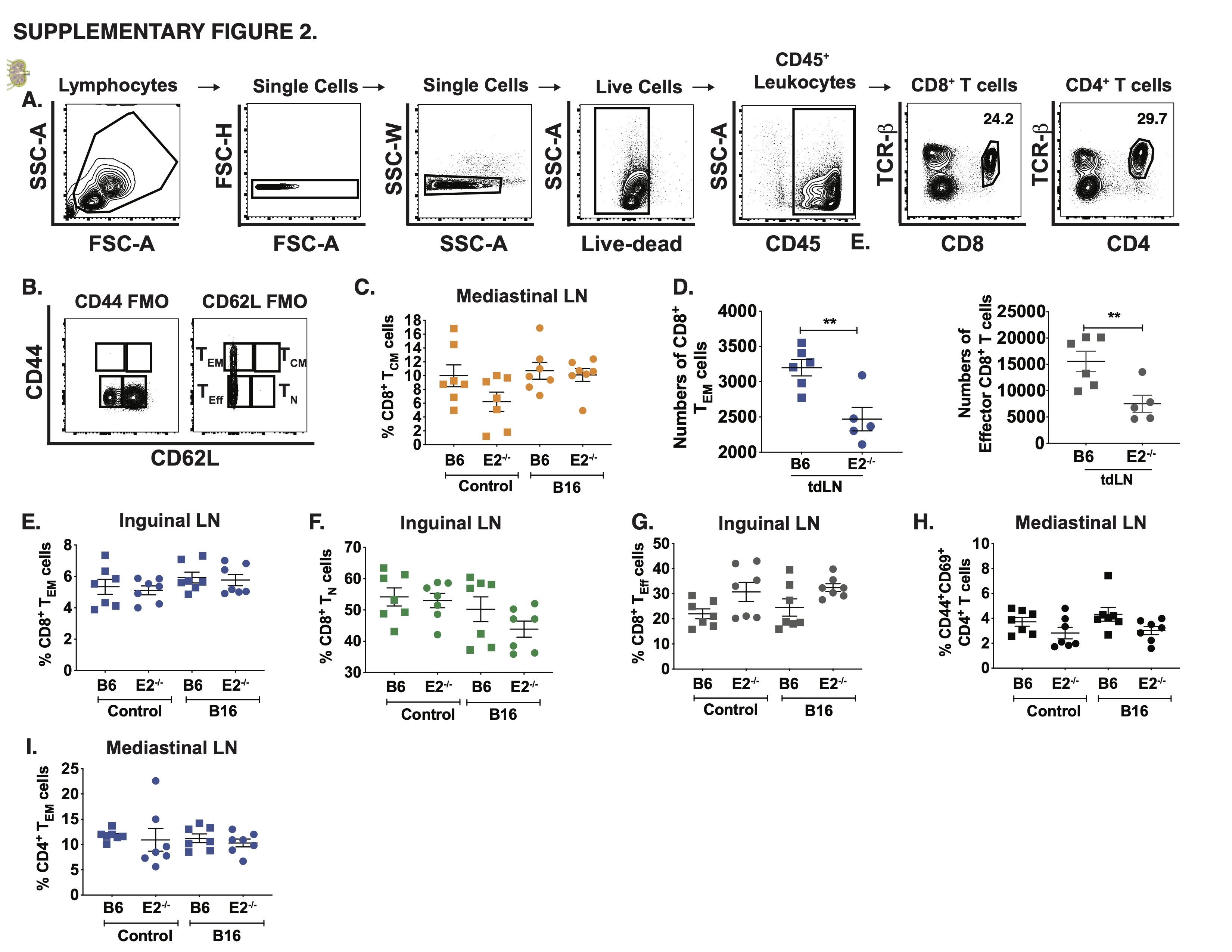

Supplement: Supplementary Figure 2 — Gating strategy for assessing CD8+ T cell activation/memory responses in the mediastinal LN and lung. CD4+TCRb+ and CD8+TCRb+ T cells were gated by lymphocytes, followed by sequential single cell gates to remove doublets (FCS-A by FSC-H; SSC-A by SSC-H), live cells (Live-dead by SSC-A), CD45+ leukocytes (CD45 by SSC-A) (A). Naive, central memory, effector memory, and effector T cells were gated utilizing fluorescence minus one (FMO) controls for CD44 and CD62L, gated on CD8+TCRb+ T cells (B). Pooled central memory CD8+ T cells frequencies in the mediastinal lymph nodes of B6 and E2-/- mice at 6 days post i.v. injection of B16F10-OVA (C). Numbers of CD8+ TEM cells in mediastinal tumor-draining LNs in B6 and E2-/- mice (D). Pooled frequencies of CD8+ effector memory (E), naive (F), and effector (G) T cells in the inguinal lymph nodes at 6 days post i.v. injection of B16F10-OVA. Pooled frequencies of CD44+CD69+CD4+ T cells, gated on live, CD45+CD4+TCRb+ T cells in the mediastinal lymph nodes of B6 and E2-/- mice at 6 days post i.v. injection of B16F10-OVA (H). Pooled frequencies of CD4+ effector memory T cells at 6 days post i.v. injection with B16F10-OVA (I). Results are expressed as mean + s.e.m. from two independent experiments. CD8 TEM numbers and CD8 Teff numbers in D were quantified by an unpaired t-test. C and E-I were quantified by One-way ANOVA. [file Image_2.jpeg]

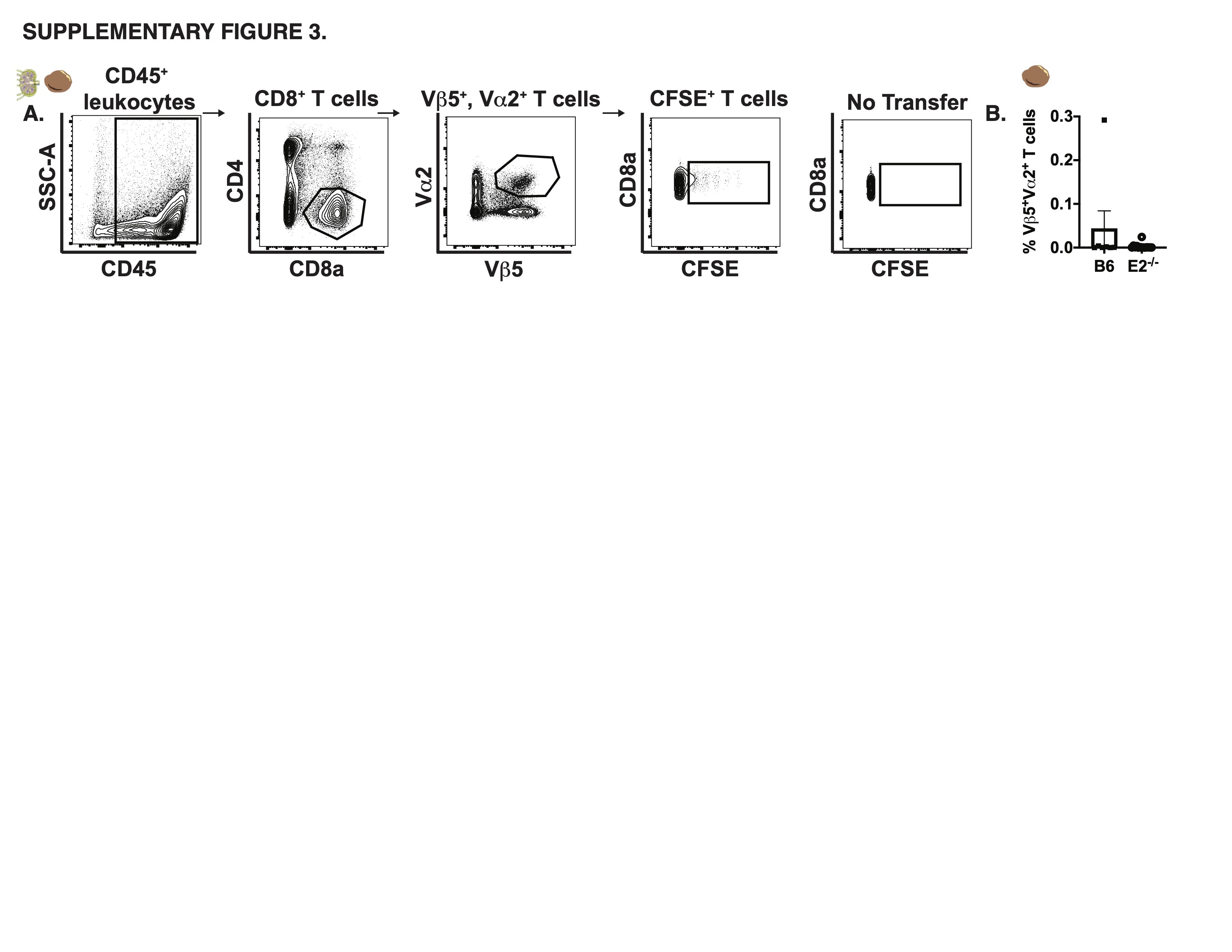

Supplement: Supplementary Figure 3 — Gating strategy for detecting adoptively-transferred OT-I CD8+ T cells. Transferred OT-I CFSE+ T cells were gated utilizing a no transfer control (A). Pooled Vb5+Va2+ T cell frequencies, gated on CD8+ T cells in B6 (n=6) and E2-/- (n=12) mice. Results are expressed as mean +/- SEM from one of three independent experiments (unpaired student’s t-test). [file Image_3.jpeg]
